# 2,2-Dichloro-*N*-(phenyl­sulfon­yl)­acetamide

**DOI:** 10.1107/S1600536808021831

**Published:** 2008-07-19

**Authors:** B. Thimme Gowda, Sabine Foro, P. G. Nirmala, B. P. Sowmya, Hartmut Fuess

**Affiliations:** a Department of Chemistry, Mangalore University, Mangalagangotri 574 199, Mangalore, India; b Institute of Materials Science, Darmstadt University of Technology, Petersenstrasse 23, D-64287 Darmstadt, Germany

## Abstract

The conformation of the N—H and C=O bonds in the title compound, C_8_H_7_Cl_2_NO_3_S, is *trans*. The benzene ring and the SO_2_—NH—CO—C group form a dihedral angle of 79.75 (8)°. Mol­ecules are connected *via* N—H⋯O hydrogen bonds to form linear supra­molecular chains.

## Related literature

For related literature, see: Gowda *et al.* (2006 ▶, 2007 ▶, 2008*a* ▶,*b* ▶); Gowda, Foro, Nirmala *et al.* (2008 ▶).
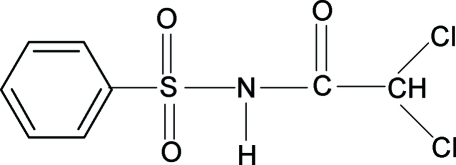


## Experimental

### 

#### Crystal data

C_8_H_7_Cl_2_NO_3_S
*M* *_r_* = 268.11
Orthorhombic, 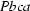

*a* = 9.669 (1) Å
*b* = 10.462 (1) Å
*c* = 21.024 (2) Å
*V* = 2126.7 (4) Å^3^
*Z* = 8
Mo *K*α radiation
μ = 0.79 mm^−1^
*T* = 299 (2) K
0.48 × 0.48 × 0.40 mm

#### Data collection

Oxford Diffraction Xcalibur diffractometer with a Sapphire CCD detector
Absorption correction: multi-scan (*CrysAlis RED*; Oxford Diffraction, 2007 ▶) *T*_min_ = 0.689, *T*_max_ = 0.728
9241 measured reflections
2156 independent reflections
1729 reflections with *I* > 2σ(*I*)
*R*_int_ = 0.038

#### Refinement

*R*[*F* ^2^ > 2σ(*F* ^2^)] = 0.045
*wR*(*F* ^2^) = 0.127
*S* = 1.13
2156 reflections
137 parameters
H-atom parameters constrained
Δρ_max_ = 0.46 e Å^−3^
Δρ_min_ = −0.61 e Å^−3^

### 

Data collection: *CrysAlis CCD* (Oxford Diffraction, 2004 ▶); cell refinement: *CrysAlis RED* (Oxford Diffraction, 2007 ▶); data reduction: *CrysAlis RED*; program(s) used to solve structure: *SHELXS97* (Sheldrick, 2008 ▶); program(s) used to refine structure: *SHELXL97* (Sheldrick, 2008 ▶); molecular graphics: *PLATON* (Spek, 2003 ▶); software used to prepare material for publication: *SHELXL97*.

## Supplementary Material

Crystal structure: contains datablocks I, global. DOI: 10.1107/S1600536808021831/tk2284sup1.cif

Structure factors: contains datablocks I. DOI: 10.1107/S1600536808021831/tk2284Isup2.hkl

Additional supplementary materials:  crystallographic information; 3D view; checkCIF report

## Figures and Tables

**Table 1:** Hydrogen-bond geometry (Å, °)

*D*—H⋯*A*	*D*—H	H⋯*A*	*D*⋯*A*	*D*—H⋯*A*
N1—H1N⋯O3^i^	0.86	2.00	2.844 (3)	166

Symmetry code: (i) 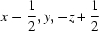.

**Fig. 1.**
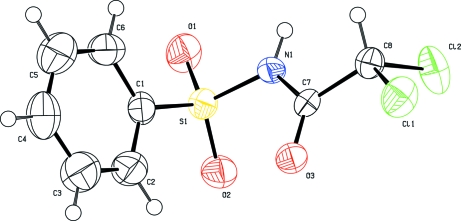
Molecular structure of (I), showing the atom labeling scheme and displacement ellipsoids at the 50% probability level.

**Fig. 2.**
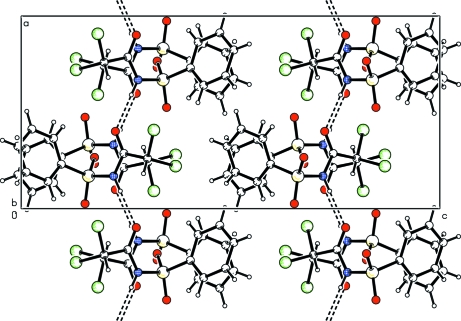
View of the molecular packing in (I) with hydrogen bonding shown as dashed lines.

## supplementary crystallographic information

### Comment

As part of a study of the substituent effects on the crystal structures of *N*-(aryl)-sulfonamides and substituted amides (Gowda *et al.*, 2006, 2007, 2008a,b; Gowda, Foro, Nirmala *et al.*, 2008), the structure of *N*-(phenylsulfonyl)-2,2-dichloroacetamide (I) has been determined. The conformation of the N—H and C=O bonds is *trans* (Fig. 1), similar to that observed in *N*-(phenylsulfonyl)-2,2,2-trimethylacetamide (Gowda *et al.*, 2008*b*), *N*-(4-methylphenylsulfonyl)- 2,2-dichloroacetamide (Gowda, Foro, Nirmala *et al.*, 2008) and (4-methylphenylsulfonyl)-2,2,2-trimethylacetamide (Gowda *et al.*, 2008*a*). Further, the bond parameters in (I) are similar to those in the aforementioned structures and in each of *N*-(aryl)-2,2-dichloroacetamides (Gowda *et al.*, 2006) and benzenesulfonamide (Gowda *et al.*, 2007). The crystal packing diagram of (I) is dominated by N—H···O hydrogen bonds (Table 1) that lead to supramolecular chains that stack to form layers, as shown in Fig. 2.

### Experimental

Compound (I) was prepared by refluxing benzenesulfonamide (0.10 mole) with an excess of dichloroacetyl chloride (0.20 mole) for about an hour on a water bath. The reaction mixture was cooled and poured into ice cold water. The resulting solid was separated, washed thoroughly with water and dissolved in warm dilute sodium hydrogen carbonate solution. Compound (I) was precipitated by acidifying the filtered solution with glacial acetic acid. It was filtered, dried and recrystallized from ethanol. Single crystals used for X-ray diffraction studies were obtained from the slow evaporation of an ethanolic solution of (I).

### Refinement

The H atoms were included in the riding model approximation with C—H = 0.93–0.98 Å and N—H = 0.86 Å, and with U(H)_iso_ = 1.2x*U*_eq_(C, N).

### Crystal data

**Table:**

C_8_H_7_Cl_2_NO_3_S	*F*_000_ = 1088
*M**_r_* = 268.11	*D*_x_ = 1.675 Mg m^−^^3^
Orthorhombic, *P**b**c**a*	Mo *K*α radiation λ = 0.71073 Å
Hall symbol: -P 2ac 2ab	Cell parameters from 3214 reflections
*a* = 9.669 (1) Å	θ = 2.8–27.9º
*b* = 10.462 (1) Å	µ = 0.79 mm^−^^1^
*c* = 21.024 (2) Å	*T* = 299 (2) K
*V* = 2126.7 (4) Å^3^	Prism, colourless
*Z* = 8	0.48 × 0.48 × 0.40 mm

### Data collection

**Table:**

Oxford Diffraction Xcalibur diffractometer with a Sapphire CCD detector	2156 independent reflections
Radiation source: fine-focus sealed tube	1729 reflections with *I* > 2σ(*I*)
Monochromator: graphite	*R*_int_ = 0.038
*T* = 299(2) K	θ_max_ = 26.4º
Rotation method data acquisition using ω and φ scans	θ_min_ = 2.9º
Absorption correction: multi-scan(CrysAlis RED; Oxford Diffraction, 2007)	*h* = −11→12
*T*_min_ = 0.689, *T*_max_ = 0.728	*k* = −12→13
9241 measured reflections	*l* = −24→25

### Refinement

**Table:**

Refinement on *F*^2^	Hydrogen site location: inferred from neighbouring sites
Least-squares matrix: full	H-atom parameters constrained
*R*[*F*^2^ > 2σ(*F*^2^)] = 0.045	*w* = 1/[σ^2^(*F*_o_^2^) + (0.0496*P*)^2^ + 3.3191*P*] where *P* = (*F*_o_^2^ + 2*F*_c_^2^)/3
*wR*(*F*^2^) = 0.127	(Δ/σ)_max_ = 0.005
*S* = 1.13	Δρ_max_ = 0.46 e Å^−^^3^
2156 reflections	Δρ_min_ = −0.61 e Å^−^^3^
137 parameters	Extinction correction: SHELXL97 (Sheldrick, 2008), Fc^*^=kFc[1+0.001xFc^2^λ^3^/sin(2θ)]^-1/4^
Primary atom site location: structure-invariant direct methods	Extinction coefficient: 0.0159 (14)
Secondary atom site location: difference Fourier map	

### Special details

**Table:**

Geometry. All e.s.d.'s (except the e.s.d. in the dihedral angle between two l.s. planes) are estimated using the full covariance matrix. The cell e.s.d.'s are taken into account individually in the estimation of e.s.d.'s in distances, angles and torsion angles; correlations between e.s.d.'s in cell parameters are only used when they are defined by crystal symmetry. An approximate (isotropic) treatment of cell e.s.d.'s is used for estimating e.s.d.'s involving l.s. planes.
Refinement. Refinement of *F*^2^ against ALL reflections. The weighted *R*-factor *wR* and goodness of fit *S* are based on *F*^2^, conventional *R*-factors *R* are based on *F*, with *F* set to zero for negative *F*^2^. The threshold expression of *F*^2^ > σ(*F*^2^) is used only for calculating *R*-factors(gt) *etc*. and is not relevant to the choice of reflections for refinement. *R*-factors based on *F*^2^ are statistically about twice as large as those based on *F*, and *R*- factors based on ALL data will be even larger.

### Fractional atomic coordinates and isotropic or equivalent isotropic displacement parameters (Å^2^)

**Table:**

	*x*	*y*	*z*	*U*_iso_*/*U*_eq_	
C1	0.2304 (3)	0.7731 (3)	0.09583 (13)	0.0374 (7)	
C2	0.3659 (4)	0.7611 (4)	0.07699 (17)	0.0546 (9)	
H2	0.4258	0.7074	0.0990	0.066*	
C3	0.4121 (4)	0.8294 (4)	0.0250 (2)	0.0686 (12)	
H3	0.5043	0.8235	0.0127	0.082*	
C4	0.3236 (5)	0.9055 (4)	−0.00819 (18)	0.0663 (11)	
H4	0.3542	0.9468	−0.0447	0.080*	
C5	0.1901 (5)	0.9217 (4)	0.0117 (2)	0.0742 (13)	
H5	0.1316	0.9773	−0.0100	0.089*	
C6	0.1421 (4)	0.8553 (4)	0.06421 (18)	0.0615 (10)	
H6	0.0515	0.8658	0.0780	0.074*	
C7	0.2865 (3)	0.8421 (3)	0.24530 (13)	0.0315 (6)	
C8	0.2599 (3)	0.9313 (3)	0.30181 (14)	0.0383 (7)	
H8	0.1815	0.9875	0.2923	0.046*	
N1	0.1690 (2)	0.7905 (2)	0.22078 (11)	0.0317 (5)	
H1N	0.0909	0.8132	0.2368	0.038*	
O1	0.0258 (2)	0.6569 (2)	0.15258 (11)	0.0517 (6)	
O2	0.2658 (2)	0.5866 (2)	0.17518 (11)	0.0474 (6)	
O3	0.40112 (19)	0.8193 (2)	0.22517 (10)	0.0425 (5)	
Cl1	0.40794 (9)	1.02379 (8)	0.31696 (4)	0.0542 (3)	
Cl2	0.22066 (11)	0.83545 (10)	0.36880 (4)	0.0626 (3)	
S1	0.16902 (7)	0.68542 (7)	0.16113 (3)	0.0355 (2)	

### Atomic displacement parameters (Å^2^)

**Table:**

	*U*^11^	*U*^22^	*U*^33^	*U*^12^	*U*^13^	*U*^23^
C1	0.0444 (16)	0.0402 (16)	0.0275 (13)	0.0016 (13)	−0.0022 (12)	−0.0021 (12)
C2	0.0468 (18)	0.069 (2)	0.0481 (19)	0.0057 (18)	0.0038 (15)	0.0114 (17)
C3	0.060 (2)	0.088 (3)	0.059 (2)	−0.001 (2)	0.0103 (19)	0.018 (2)
C4	0.086 (3)	0.073 (3)	0.0405 (19)	−0.007 (2)	0.0074 (19)	0.0124 (18)
C5	0.092 (3)	0.074 (3)	0.057 (2)	0.024 (3)	−0.002 (2)	0.024 (2)
C6	0.059 (2)	0.077 (3)	0.0480 (19)	0.025 (2)	0.0045 (17)	0.0120 (18)
C7	0.0317 (14)	0.0342 (13)	0.0285 (13)	0.0023 (11)	−0.0048 (11)	0.0012 (11)
C8	0.0379 (14)	0.0392 (16)	0.0377 (15)	0.0014 (13)	−0.0053 (12)	−0.0059 (12)
N1	0.0231 (10)	0.0409 (13)	0.0310 (11)	0.0013 (9)	0.0000 (9)	−0.0042 (10)
O1	0.0397 (12)	0.0637 (15)	0.0517 (13)	−0.0144 (11)	−0.0074 (10)	−0.0088 (11)
O2	0.0553 (13)	0.0384 (12)	0.0486 (12)	0.0084 (10)	0.0025 (11)	0.0014 (10)
O3	0.0247 (10)	0.0621 (14)	0.0407 (11)	0.0015 (9)	−0.0020 (8)	−0.0080 (10)
Cl1	0.0589 (5)	0.0460 (5)	0.0577 (5)	−0.0117 (4)	−0.0141 (4)	−0.0070 (4)
Cl2	0.0783 (7)	0.0726 (6)	0.0369 (4)	−0.0188 (5)	0.0138 (4)	−0.0085 (4)
S1	0.0355 (4)	0.0372 (4)	0.0338 (4)	−0.0009 (3)	−0.0024 (3)	−0.0022 (3)

### Geometric parameters (Å, °)

**Table:**

C1—C2	1.375 (5)	C6—H6	0.9300
C1—C6	1.382 (5)	C7—O3	1.210 (3)
C1—S1	1.755 (3)	C7—N1	1.359 (3)
C2—C3	1.380 (5)	C7—C8	1.533 (4)
C2—H2	0.9300	C8—Cl1	1.757 (3)
C3—C4	1.361 (6)	C8—Cl2	1.770 (3)
C3—H3	0.9300	C8—H8	0.9800
C4—C5	1.368 (6)	N1—S1	1.668 (2)
C4—H4	0.9300	N1—H1N	0.8600
C5—C6	1.384 (6)	O1—S1	1.428 (2)
C5—H5	0.9300	O2—S1	1.425 (2)
			
C2—C1—C6	120.5 (3)	O3—C7—N1	123.7 (3)
C2—C1—S1	120.0 (2)	O3—C7—C8	123.0 (2)
C6—C1—S1	119.5 (3)	N1—C7—C8	113.3 (2)
C1—C2—C3	119.3 (3)	C7—C8—Cl1	109.8 (2)
C1—C2—H2	120.3	C7—C8—Cl2	107.9 (2)
C3—C2—H2	120.3	Cl1—C8—Cl2	110.02 (16)
C4—C3—C2	120.4 (4)	C7—C8—H8	109.7
C4—C3—H3	119.8	Cl1—C8—H8	109.7
C2—C3—H3	119.8	Cl2—C8—H8	109.7
C3—C4—C5	120.6 (4)	C7—N1—S1	123.15 (19)
C3—C4—H4	119.7	C7—N1—H1N	118.4
C5—C4—H4	119.7	S1—N1—H1N	118.4
C4—C5—C6	119.8 (4)	O2—S1—O1	120.73 (15)
C4—C5—H5	120.1	O2—S1—N1	108.81 (13)
C6—C5—H5	120.1	O1—S1—N1	103.44 (13)
C1—C6—C5	119.3 (4)	O2—S1—C1	108.64 (14)
C1—C6—H6	120.4	O1—S1—C1	109.80 (15)
C5—C6—H6	120.4	N1—S1—C1	104.10 (13)
			
C6—C1—C2—C3	−1.7 (6)	O3—C7—N1—S1	2.0 (4)
S1—C1—C2—C3	179.2 (3)	C8—C7—N1—S1	−177.32 (19)
C1—C2—C3—C4	−1.7 (7)	C7—N1—S1—O2	49.4 (3)
C2—C3—C4—C5	4.2 (7)	C7—N1—S1—O1	178.9 (2)
C3—C4—C5—C6	−3.3 (7)	C7—N1—S1—C1	−66.3 (3)
C2—C1—C6—C5	2.5 (6)	C2—C1—S1—O2	−15.1 (3)
S1—C1—C6—C5	−178.3 (3)	C6—C1—S1—O2	165.7 (3)
C4—C5—C6—C1	0.0 (7)	C2—C1—S1—O1	−149.1 (3)
O3—C7—C8—Cl1	15.7 (4)	C6—C1—S1—O1	31.7 (3)
N1—C7—C8—Cl1	−164.9 (2)	C2—C1—S1—N1	100.7 (3)
O3—C7—C8—Cl2	−104.2 (3)	C6—C1—S1—N1	−78.5 (3)
N1—C7—C8—Cl2	75.2 (3)		

### Hydrogen-bond geometry (Å, °)

**Table:**

*D*—H···*A*	*D*—H	H···*A*	*D*···*A*	*D*—H···*A*
N1—H1N···O3^i^	0.86	2.00	2.844 (3)	166

Symmetry codes: (i) *x*−1/2, *y*, −*z*+1/2.
